# Retrospective auditory cues can improve detection of near-threshold visual targets

**DOI:** 10.1038/s41598-019-55261-0

**Published:** 2019-12-12

**Authors:** Daphné Rimsky-Robert, Viola Störmer, Jérôme Sackur, Claire Sergent

**Affiliations:** 1 Integrative Neuroscience and Cognition Center - UMR 8002 CNRS/Université Paris Descartes, Paris, France; 2 Department of Psychology & Neuroscience Graduate Program, University of California, San Diego, USA; 3 LSCP, École des Hautes Études en Sciences Sociales, Paris, France

**Keywords:** Attention, Consciousness, Perception

## Abstract

Recent studies have demonstrated that visually cueing attention towards a stimulus location after its disappearance can facilitate visual processing of the target and increase task performance. Here, we tested whether such retro-cueing effects can also occur across different sensory modalities, as cross-modal facilitation has been shown in pre-cueing studies using auditory stimuli prior to the onset of a visual target. In the present study, participants detected low-contrast Gabor patches in a speeded response task. These patches were presented in the left or right visual periphery, preceded or followed by a lateralized and task-irrelevant sound at 4 stimulus-onset asynchronies (SOA; −600 ms, −150 ms, +150 ms, +450 ms). We found that pre-cueing at the −150 ms SOA led to a general increase in detection performance irrespective of the sound’s location relative to the target. On top of this temporal effect, sound-cues also had a spatially specific effect, with further improvement when cue and target originated from the same location. Critically, the temporal effect was absent, but the spatial effect was present in the short-SOA retro-cueing condition (+150 ms). Drift-diffusion analysis of the response time distributions allowed us to better characterize the evidenced effects. Overall, our results show that sounds can facilitate visual processing, both pre- and retro-actively, indicative of a flexible and multisensory attentional system that underlies our conscious visual experience.

## Introduction

What mechanisms lay the ground for conscious experience? As this question remains debated to this day, two radically different accounts aim at solving the issue. On the one hand it is argued that consciousness arises from local recurrent loops of information in the sensory cortices, and that top-down selection processes such as attention gate reportability of that information, rather than perceptual awareness itself^1–3^. On the other hand, other researchers argue that perceptual awareness is the result of information transfer across domains, and that a broad network of regions beyond the sensory cortices is involved^4,5^. A key difference between these two theories is that, in the first case, the conscious or non-conscious fate of a sensory input is decided within sensory areas before any involvement of supra-modal areas. This means that whether a stimulus becomes conscious or not is determined within the first 100–150 ms following its presentation^6^. In the latter case, supra-modal or other sensory cortices are pivotal for conscious experience, and perceptual awareness is not dependent only on local recurrent information transfer within a single sensory modality.

Recent studies^7,8^ used an attentional spatial cueing paradigm^9^ in which a cue could appear before or after the appearance of a target event to test the broadcasting vs. local feedback account of conscious perception: indeed, if conscious perception correlates with the broadcast of sensory information via top-down attention across the brain, then retrospectively orienting attention towards the sensory trace of a target could potentially promote initially unseen stimuli into consciousness. In contrast, if conscious perception is decided during the initial sensory processing steps, as assumed by the local feedback account, a retro-cue that directs attention to the target retroactively should not influence whether the stimulus is consciously perceived or not. Previous research tested these two predictions by asking participants to judge the orientation of low-contrast Gabor patches while spatial visual retro-cues attracted attention either to the target’s past location or to the opposite location. These studies showed that such retrospective cueing of exogenous spatial attention facilitated conscious perception of the past target – similar to previously observed pre-cueing effects where the cue is presented at the target location prior to its onset^9^. This effect was called “retro-perception”^10^, and highlighted the existence of temporal flexibility in the processes allowing for conscious access, while providing evidence for the importance of attention as a gating mechanism for conscious experience. However, since these studies used spatial cues within the same modality as the target, it cannot be excluded that this retro-perception effect was at least partly due to a low-level, visual interaction between the retrospective cue and the visual trace of the target, rather than to top-down attentional involvement^11^.

In order to address this question we devised an experiment in which the target and the retrospective cues where presented in different sensory modalities, in order to minimize direct local interactions between experimental stimuli within the sensory cortex. In particular, we used auditory cues and visual targets because they are initially processed in distinct cerebral regions. While there exists evidence that these could interact through rapid direct pathways between the sensory cortices with no involvement of supra-modal structures^12^, this can be avoided by using stimulus-onset asynchronies (SOAs) above 100 ms^13^. Many previous studies have shown that hearing a salient sound can facilitate processing of subsequently presented visual stimuli at the same location^14^. These cross-modal effects of attention have been shown to not only speed response times, but also increase visual sensitivity^15,16^. For example, pre-cueing the occurrence of a visual target with a peripheral sound has been shown to increase participants’ detection rate of visual stimuli near the perceptual threshold^15^, facilitate the discrimination of visual targets^17^, enhance perceived contrast of the target^18^, or accelerate the perceived timing of a visual event^19^. These behavioral effects are accompanied by an increase in early neural responses to the visual target over the occipital cortex contralateral to the side of presentation^20^. These results suggest that cross-modal orienting of attention can alter sensory processing in the visual cortex. However, in all these studies, the auditory cue preceded the target. Here we wanted to test whether auditory retro-cues could also affect perception of near-threshold visual stimuli– similar to what has been shown for visual retro-cues^7^. While retro-cueing studies exist on cross-modal perception, most are focused on the effects of exogenous cues on supraliminal items held in working memory^21,22^: here, we did not aim to alter actively maintained representations, but rather determine whether exogenously retro-cued attention could elicit, across sensory modalities, the report of stimuli that would have otherwise remained unseen.

Additionally, we were interested in assessing the effect of cross-modal retrospective cues not only on perceptual accuracy but also on response times. While the influence of a pre-cue on speeded responses has been studied extensively, showing acceleration of response times with valid pre-cueing^23–30^, retro-perception has not yet been examined in this manner. If auditory retro-cues can influence whether visual stimuli are consciously perceived, we expect that they will also influence how long it takes to report their presence. However, predictions on the direction of the effect are not entirely straightforward: if retro-cueing improves perception it might speed up detection response time, in accordance with the effect observed for pre-cues. But one might also predict the contrary: if, as suggested by previous studies, retrospective cues retrospectively promote unseen stimuli into awareness, then response times on those trials could be delayed since response time might be then time-locked to the retro-cue rather than time-locked to the target presentation itself.

We thus designed an experiment aiming to extend retro-perception to a cross-modal setting in order to generalize the phenomenon beyond the visual modality, as well as examine its effect on perceptual decision-making. The experiment was a variation of an original paradigm by McDonald and colleagues showing the effect of exogenous auditory pre-cueing on visual perception^31,32^. Participants had to press a button as soon as they detected the presence of a visual Gabor patch to the left or to the right of fixation. We investigated how detection sensitivity and response time to this visual target was influenced by a salient auditory cue that could emanate from the same location as the target (congruent cue) or from the opposite location (incongruent cue) either before or, critically, after the visual stimulus. As in McDonald and colleagues’ original experiment, the location of the auditory cue was random relative to the target’s location, so that the cue only mobilized reflexive, exogenous attention.

While existing retro-cueing studies on working memory in cross-modal paradigms evidence strongest effects after 300 ms, retro-perception experiments are focused on shorter SOAs, because they are designed to affect perceptual processing. We thus used 4 different SOAs: −600 ms and −150 ms in pre-cueing, and +150 ms and +450 ms in retro-cueing. We hypothesized that at short SOAs (−150 ms and +150 ms), cue validity should influence performance. Whether the −600 ms SOAs should display inhibition of return or no effect at all was undetermined, as this effect is more context-dependent in cross-modal settings^33^. Finally, the original study on retro-perception showed that a positive cue-congruency effect was still present for retro-cues at 400 ms, although it was reduced compared to effects observed for retro-cues at 100 or 200 ms^7,8^. Including a +450 ms SOA in the present study allowed us to assess the time course of potential cross-modal retro-perception effects, while keeping a constant distance of 300 ms between SOAs of interest.

## Results

### D prime

**Figure 1:**
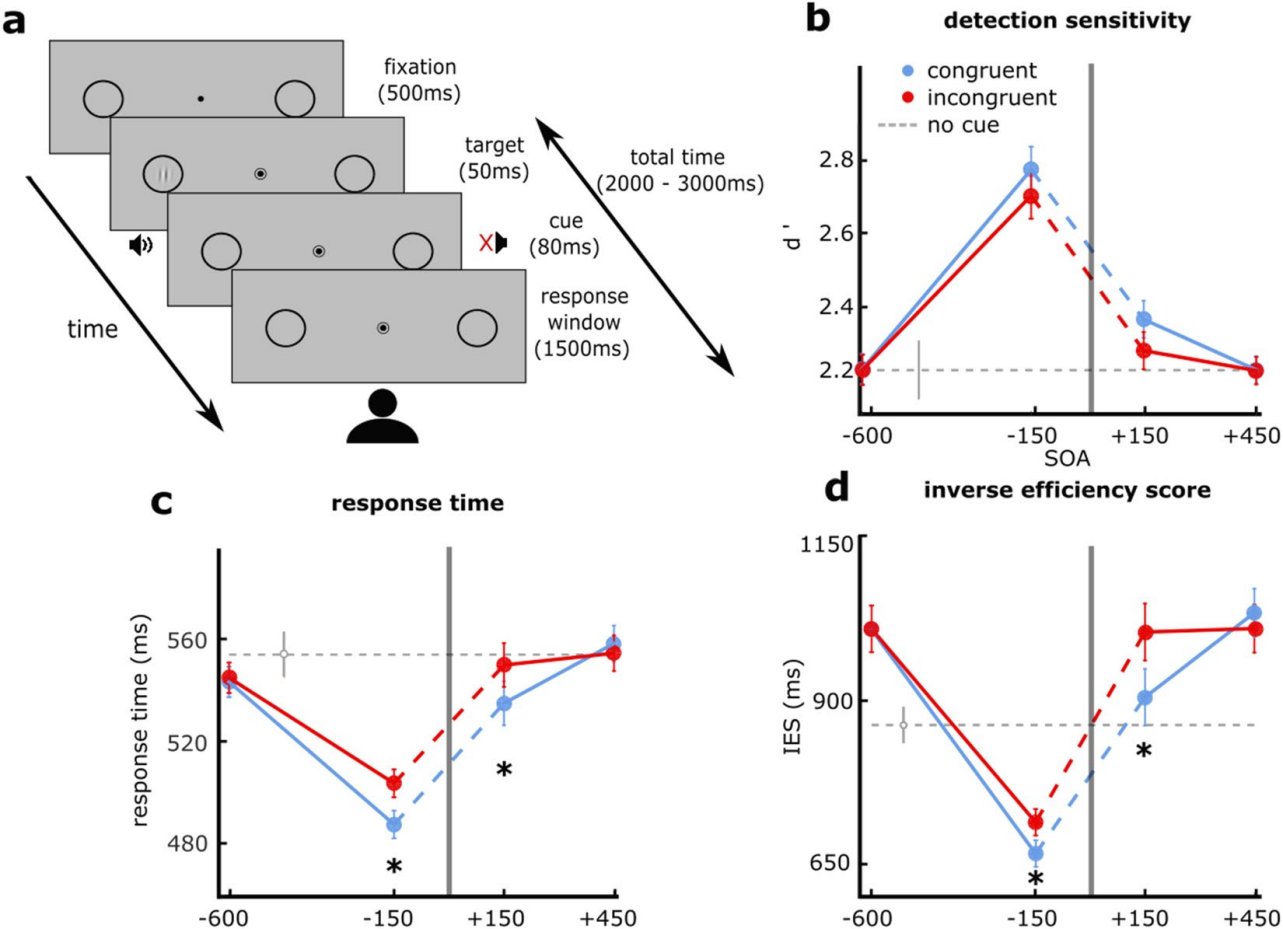
Task design and main results. On each trial, participants had to detect a low-contrast Gabor patch that could appear in one of two placeholders on the screen with a probability of 2/3. The jittered delays preceding and following the appearance of the target were 1–1.5 s. Before or after the target, a spatially non-informative auditory cue could appear at one of four SOAs: −600 ms, −150 ms, +150 ms, and +450 ms. (**a**) Detection sensitivity: colored lines represent mean d’ for congruent (blue) and incongruent (red) trials at each SOA. The dashed line is performance in the no-cue condition. The grey line represents the time of target presentation. Error bars are standard errors of the mean difference across observers, except for the no-cue condition, where it is the standard error of the mean. (**b**) Response times: colored lines represent averaged median RT for congruent and incongruent trials at each SOA across participants. Error bars are standard errors of the mean difference across observers, except for the no-cue condition, where it is the standard error of the mean. (**c**) Inverse efficiency scores: colored lines represent averaged mean IES for congruent and incongruent trials at each SOA across participants. Error bars are standard errors of the mean difference across observers, except for the no-cue condition, where it is the standard error of the mean. (**d**) Stars represent statistical significance as indicated in the main text.

Figure 1b shows the results on detection sensitivity (d’) separately for the different types of cues as a function of SOA. Overall detection performance increased sharply when the sound cue occurred just before the visual stimulus (−150 ms SOA) compared to sounds presented earlier, i.e. 600 ms before the target, and also compared to the no-cue condition. This was confirmed by a 2 (congruency) × 4 (SOAs) repeated measures ANOVA which revealed a significant main effect of SOA on detection sensitivity (F(1.83,34.67) = 14.03, p < 0.001, generalized eta squared g-η² = 0.081). Furthermore, 10,000 bootstrapped confidence intervals of the mean difference between the cued conditions (either congruent or incongruent) and no-cue conditions revealed a significant difference in mean sensitivity for the short pre-cue SOA (−150 ms), but no other (−600 ms: CI = [−0.26 0.28], −150 ms: CI = [0.26 0.74], +150 ms: CI = [−0.14 0.31], +450 ms: CI = [−0.23 0.21]), suggesting that this effect was indeed specific for the short-SOA pre-cueing condition. This observation is in line with previous research showing that pre-cueing a visual stimulus with an auditory cue globally improves participants’ performance regardless of location, particularly in visual search^34–36^. Interestingly this effect was not symmetrical: it was either absent or reduced when the cue came after the stimulus (+150 ms SOA).

On top of this strong modulation by SOA, spatial congruency between target and cue also affected detection sensitivity: visual detection sensitivity improved when the exogenous auditory cue appeared at the same side as the target (congruent) compared to the opposite side (incongruent) both for pre- and retro-cues at short SOAs. The ANOVA confirmed a significant main effect of congruency (F(1,19) = 4.53, p = 0.046, g-η² = 0.001), but no interaction between congruency and SOA (F(3,57) = 0.72, p = 0.5, g-η² = 0.001). Pairwise comparisons were not reported as the ANOVA did not reveal an interaction between factors. Overall, these data replicate what was evidenced in the literature, showing that cross-modal pre-cues can enhance target detection^15,37^. Based on d’ alone, the effects of spatial congruency on target detection were subtle, but present. The relatively small magnitude of the effects is expected, particularly in a speeded-response task, and consistent with other studies showing smaller effects of cross-modal relative to unimodal cues^38,39^.

### Response time

Response time data for Hit trials was analyzed (Fig. 1c) across participants using a generalized mixed-effects model (GLMM) with an inverse Gaussian parent distribution, including SOA, congruency and their interaction as fixed effects and participant number as a random effect on the intercept. The profile of response time observed in the different conditions seemed to mirror what was found for detection sensitivity. Post-hoc confidence intervals of the mean difference between cued and uncued conditions revealed a significant difference in response time for the short pre- and post-cued (−150 ms and +150 ms) SOAs (−600 ms: CI = [−31.35–7.14], −150 ms: CI = [−77.26–−42.63], +150 ms: CI = [−25.02–0.91], +450 ms: CI = [−10.73 12.10]). This first analysis of response time data showed that pre- and post-cueing within a short time-window, regardless of congruency, induced a shortening of response time.

The GLMM revealed a significant modulation of response time by SOA (χ² (3) = 381.55, p < 10^−15^) as well as a significant decrease in response time with congruency (χ²(1) = 8.19, p = 0.004), and an interaction between these factors (χ²(3) = 20.536, p = 0.0001). Interestingly, the effect of congruency is evidenced more clearly here than with d’. Still, both patterns go in the same direction, suggesting that cue location increases detection rate and reduces response time. For each SOA we further assessed Tukey’s all-pair HSD test on the difference between congruent and incongruent condition, which was the contrast of interest from our initial hypotheses. The corresponding 95% confidence intervals of the mean RT difference between congruent and incongruent conditions suggest an effect of congruence both at the short pre and retro-cue SOAs, but not at the longer SOAs (−600 ms = [−18.07, 14.20], −150 ms = [1.33, 37.81], +150 ms = [9.38, 37.09], +450 ms = [−31.66, 2.99]).

Since the patterns of results for d’ and RTs both go in the same direction, this suggests that both reflect a general improvement of processing efficiency with spatially congruent cues. We thus conducted a third analysis that combined both measures. We calculated the inverse efficiency score (IES)^40,41^ for each participant in all SOA and congruency conditions (Fig. 1d).

The GLMM revealed a significant effect of SOA (χ²(3) = 1755.44, p < 10^−15^) and congruency (χ²(1) = 31.17, p < 10^−7^), and an interaction between these factors (χ²(3) = 30.90, p < 10^−7^). 95% confidence intervals (corrected Tukey’s all pair HSD test) indicate a significant reduction of the IES with congruent cues at −150 ms (CI = [22.57 57.85]) and +150 ms (CI = [42.81 91.70]), but no other SOA (−600 ms = [−25.05 16.80], +450 ms = [−53.34 5.92]).

Last, we estimated the Spearman’s correlation coefficient between effect sizes in IES for the contrast “congruent vs. incongruent” between the two SOAs of interest (−150 ms and +150 ms), across participants. There was indeed a significant positive correlation between the size of the pre and retro-cueing effect across participants (R = 0.47, p = 0.04), possibly suggesting that both effects rely at least partly on similar mechanisms.

As far as congruency is concerned, the effect of cueing were primarily present at the short pre- and post-cued SOAs (−150 ms and +150 ms) in all our measures: d’, response time, and IES.

### Model fitting diagnostics

**Figure 2:**
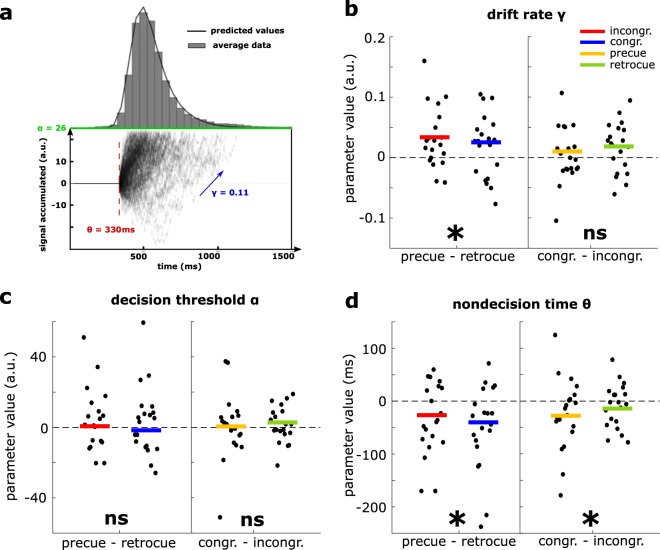
Drift-diffusion analysis of RT. The shifted-Wald distribution can be used to describe signal accumulating to a threshold α, at rate γ, with θ representing the time lapsed outside this decision process. Participant-wise distributions were fitted across all cue and target-present trials, and group-average parameters were retrieved (colored values). 10,000 trials were simulated using the group parameters. A subset of these trials is displayed, their random walk with drift is shown as signal accumulated over time. Crossing the threshold α results in a response, with a corresponding RT. The simulated distribution from group parameters (black line) is shown over real data (grey bars shown the average RT distribution across participants). (**a**) The mean difference in the drift rate γ is shown for each participant (black dots) for the contrast pre-cue minus post-cue, split according to congruency (Left Panel, group average in yellow) and congruent minus incongruent split according to SOA (Right Panel, group average in green). Significance of the two-way RM ANOVA is indicated at the top left with a star, or the mention ns (not significant). (**b**) Group analysis of the decision threshold α as described above. (**c**) Group analysis of the non-decision time θ as described above (**d**).

Because response time data are positively skewed, common summary statistics such as means poorly describe potential effects of an experiment on the tail of the distribution^42^. While GLMMs can prove to be very informative, more complex analyses can help provide a more refined description of the processes underlying the observed RT distributions. We thus fitted a shifted-Wald distribution using MLE as described in the Methods and illustrated in Fig. 2a. In brief the parameters of interest used in the model are *γ*, the drift rate, which is thought to reflect the quality of the information being processed, *α*, the decision threshold, which reflects cognitive control (e.g. inhibitory control over the button press), and *θ*, the non-decision time, which is affected when motor demands for responding become increasingly difficult, or the course of perceptual processing or motor preparation is altered^43,44^.

For this planned analysis, we only included SOAs where an effect of congruency on response times and accuracy could be observed in our initial analysis on RTs, i.e. the two short SOAs that were the focus of our initial hypotheses. This left us with S = 2 SOAs, C = 2 congruence levels, P = 20 participants, leading to 2 × 2 × 20 = 80 distributions (or experimental cells) to be fitted individually with an average number of trials of 55 ± 10, which is enough according to the simulations run in^43^.

We followed the 3-step model-fitting goodness of fit checks recommended in^43^. The QQ plot in the Supplementary Fig. 1.A shows no systematic misfit of the predicted values against the actual RT data, but highlights the presence of several outlying data points that have very large values. The decile residual distribution plot is shown in Supplementary Fig. 1.B. Because the data is positive and right-skewed, we should expect the variance in the residuals to increase with variance in the data and decile number. To partially account for this bias when performing goodness of fits checks, the residual standard deviations were standardized by the shifted-Wald standard deviation ($SD(X)=\,\sqrt{\alpha /{\gamma }^{3}}$). Here, the plot shows a good ordering of the deciles, apart from a few outlying data-points in extreme RT values. The last goodness of fit plot (Supplementary Fig. 1.C) shows the sum of standardized residuals by experimental cell Δ, and denotes the 95% quantile range of these values as well as its mean $\bar{\Delta }$, and standard deviation σ. The reported value ${{\rm{\rho }}}_{\Delta {\rm{\sigma }}}$ is the Pearson correlation coefficient between Δ and SD, which measures how efficient SD is as a standardization statistic. Values of $\bar{\Delta }$ and ${{\rm{\rho }}}_{\Delta {\rm{\sigma }}}$ should be as close to zero as possible, a requirement that is reasonable here for $\bar{\Delta }$ and ${{\rm{\rho }}}_{\Delta {\rm{\sigma }}}$. Overall, we meet the requirements for these three goodness of fit checks, although they reveal some data-points are outlying.

### Model fitting results

Parameters α, γ and θ were retrieved after model fitting and goodness of fit checks for each participant and condition (SOA, congruency). Each parameter was assessed for statistical significance using repeated measures ANOVA across participants for all conditions and their interactions. For the drift rate γ (Fig. 2b), this analysis revealed a significant main effect of SOA in favor of a larger drift rate in pre-cueing compared to retro-cueing (F(1,19) = 9.29, p = 0.007, g-η² = 0.100) but no significant effect of congruency (F(1,19) = 3.22, p = 0.09, g-η² = 0.017) or their interaction (F(1,19) = 0.65, p = 0.43, g-η² = 0.007). For the threshold parameter α (Fig. 2c), no predictor variable significantly affected its value (SOA F(1,19) = 1.34, p = 0.26, g-η² = 0.022; congruency F(1,19) = 0.26, p = 0.61, g-η² = 0.002; interaction F(1,19) = 0.03,p = 0.87, g-η² = 0.0003). For the non-decision time θ (Fig. 2d), a significant main effect of SOA (F(1,19) = 9.80, p = 0.006, g-η² = 0.104) and congruency (F(1,19) = 4.63, p = 0.045, g-η² = 0.019) were evidenced, but no interaction (F(1,19) = 0.61, p = 0.44, g-η² = 0.004).

Overall, these results allow us to specify the origin of the effects observed on RTs: the SOA affected both the drift rate and the non-decision time, whereas congruency only affected the non-decision time. The decision boundary was unaffected by the experimental manipulation.

## Discussion

Besides improving perception of future visual events at a location, can spatialized sounds help revive past events that would otherwise not have been seen? In this study we tested whether exogenous cross-modal attention can affect perception retrospectively. To test this, we adapted an original study conducted by McDonald *et al*. which demonstrated the existence of cross-modal effects in the case of pre-cued attention^31,32^. The present study replicated their original effect by showing that a non-informative but spatially congruent auditory pre-cue increases detection sensitivity to a visual stimulus. The use of several pre- and retro-cueing SOAs, as well as a no-cue condition, allowed us to show that there were two phenomena at play in cross-modal cueing effects. One was a general “arousal” effect that was independent of cue location but sensitive to the cue-target timing: pre-cueing shortly before the target (−150 ms) induced a general increase in perceptual sensitivity and a response time reduction regardless of location congruency. This effect was absent when the pre-cue was presented long before the target (−600 ms) and was abolished for retro-cues, irrespective of the SOA (+150 ms and +450 ms). This effect is thus sensitive to the temporal sequence of stimulus presentation, and in line with previous studies conducted in pre-cued visual search on cross-modal influences on perception^34–36^. Another aspect of the cue effect is its sensitivity to cue location: when cue and target appeared at the same location, detection sensitivity was increased and RTs were decreased for both short SOA conditions (−150 ms and/or +150 ms). Importantly, while the spatial congruency effects were only observed for short SOAs, they did not appear to be influenced by whether the cue appeared before or after the target. Interestingly, congruency effect sizes were correlated between the pre- and retro-cueing conditions when using the inverse efficiency score which accounts for both response time and accuracy. This provides some initial evidence suggesting that these effects may be related to a single underlying process. However, it should be acknowledged that the spatial effects observed in these studies were relatively small, both for pre and retro-cueing conditions, compared to previously reported cross-modal pre-cueing effects^20^. This might be due to the fact that the auditory and visual stimuli did not emanate from exactly the same location in space (the loudspeakers were on the side of the screen). In future studies, it would be important to replicate these effects in conditions where cross-modal pre-cuing is known to be particularly strong, notably by having the visual stimuli at the exact location of the loudspeakers^15^.

A more fine-grained analysis that focused on the response time distributions revealed that the non-spatial effect of pre-cueing at the short SOA corresponded – at least in part – to an increase in the drift rate, which is thought to reflect an improved quality of sensory information. The effect on the drift-rate is consistent with the increase in perceptual sensitivity (d’) in the short pre-cueing condition seen in Fig. 1.B, and may be the result of a general effect of well-timed sounds on visual perception, as has been reported in several studies^34–36^. The modeling analysis additionally showed that pre-cueing reduced the non-decision time as well when compared to retro-cueing. Generally, it is considered that this delay is related to perceptual encoding, presumably prior to process of evidence accumulation, as well as motor preparation and response execution time following the decision process. Here, since the response requirements are identical in all conditions (same button press), it seems difficult to attribute the effects of our experimental conditions to response execution. Rather, we think these results can be interpreted in terms of alertness, whereby the occurrence of a pre-cue may have given a “headstart” to motor preparation and/or primed the sensory system for target appearance in the near future^45^. Alternatively, it may have been the case that participants learned something about the timing of the auditory-visual sequence and build expectations such that the sound itself triggered an expectation of a visual stimulus to appear 150 ms afterwards (presumably, this would be an optimal strategy, as a failure to detect the stimulus would simply involve a switch to expecting the stimulus to appear 600 ms after cue-onset). However, given the various SOA conditions as well as a no-cue condition that were all randomly intermixed, such temporal expectation effects appear rather unlikely.

Spatial congruency of the cue affected the non-decision time alone. The model used to analyze response time distributions does not allow to completely disentangle motor preparation from stimulus encoding. However, while the presence of a cue can be expected to influence motor preparation, it is difficult to imagine how its location with regards to a visual stimulus would have an effect purely on motor planning when the elicited response does not depend on the cue’s location (simple detection). According to previous literature on retro-perception, spatially congruent cues lower the threshold for conscious access, even retrospectively, thus triggering conscious access to targets that would have been unreported otherwise^7,8^. It is plausible in this case that congruent retro-cues affected the perceptual component of stimulus processing rather than motor-related components, which is generally compatible with our hypothesis that these auditory retro-cues affected the probability of conscious access. We had initially hypothesized that retro-perception may increase response times because conscious processing would be initiated at a later time. In this experiment, we found the opposite: spatially congruent cues actually shortened them, and in fact, this was also the case in previous studies on retro-perception^7^. In the context of such accumulator models for a detection task, triggering conscious access may correspond to the onset of evidence accumulation. In this case, retro-cues may boost processing in more ambiguous trials at the perceptual level and hasten the onset of the decision process, which is not affected in itself. These results are compatible with previous studies on retro-perception, which showed that retro-cueing of this kind led to an increased chance of conscious perception without better information about the target stimulus^8^.

Broadly, our results support a theory in which conscious perception is not just determined by local recurrent feedback within a sensory modality, but is modulated by inputs across sensory areas, possibly via a general, supra-modal attention system. While previous research showed that orienting spatial attention to a sound can improve target detection of a subsequent co-localized stimulus, we here showed that such cross-modal benefits in perceptual sensitivity can also operate retrospectively. What may be the mechanisms underlying these behavioral benefits? One possibility is that by allocating attention to the cued location after the visual stimulus disappeared strengthens the sensory trace of the visual target by boosting visual-cortical activity at that location thanks to the occurrence of the sound. This interpretation is consistent with neurophysiological findings indicating that a sound alone – without any visual inputs – can trigger a response in visual areas that is spatially specific. In particular, it has been found that a peripheral sound activates visual cortex contralateral to its location, even in purely auditory tasks^31,32,46^. Thus, the post-cue in the current study may re-activate or enhance the activity triggered by the visual stimulus, ultimately pushing it into consciousness. A further line of research may investigate how cross-modal retro-perception affects the outcome of sensory processing besides simple detection, as there exists evidence that cross-modal retro-cues can alter other perceptual processes, such as time-order judgements for supraliminal stimuli^47^. The fact that such a change in target detectability occurs across modalities and across different time scales demonstrates how different inputs can flexibly shape our perceptual experience, ultimately determining our conscious visual experience.

## Methods

### Participants

Based on previous experiments on retro-perception^7,8^, the number of participants was fixed to 20 prior to the experiment. 26 healthy participants were recruited, out of which 6 were excluded (failure to find the perceptual threshold (4), performance at chance in the main task (1) or the participant left before completing the experiment (1)). The remaining participants (18 females) had a mean age of 25 y-o ±3.86. All participants gave informed consent in writing prior to participation, and the Université Paris Descartes Review Board CER-Paris Descartes (Research Ethics Committee) approved the protocols for the study in accordance with French regulations and the Declaration of Helsinki. Participants received a compensation of 10€ per hour of their time.

### Materials

The experiment was a variation on an original paradigm used in several instances^31,32^ and conducted in a dimly lit room. The background sound level in the room was 45 dB, which is expected for a quiet room with a running computer. Participants sat 110 cm away from an extra-large screen (OLED SAMSUNG Smart TV), 125 cm large by 71 cm tall. Screen resolution was 1920 × 1080 px and refresh rate was 60 Hz. Luminance of the screen background was at around 18.2 cd/m² on average. Loudspeakers were mounted on the sides of the screen, their center measured at 35° from the fixation point at the center of the screen. Stimuli were generated and responses recorded using the Psychophysics Toolbox for Matlab^48^. Eye fixation was monitored using an Eyetribe tracking device (The Eye Tribe, Copenhagen).

### Stimuli and procedure

Participants were asked to fixate a small dot (black, 0.2° diameter) at the center of the screen (Fig. 1a). A concentric circle (black, 0.4° diameter) appeared around the dot at the beginning of a trial, and disappeared at the end of the trial. During each trial two placeholders were permanently present on both sides of the screen, indicating the two locations where a visual target could appear. These placeholders were black circles with an 8° diameter. Visual targets were vertical Gabor patches (placeholder external edge at 30° eccentricity, center at 26°) with luminance variations of 1.5 cycle per degree modulated by a Gaussian envelop of 2.2° full width half maximum. These visual targets appeared randomly within either the left or right placeholder and lasted ~53 ms. The auditory cue was a 78 dB burst of broadband (500–15,000 Hz) pink noise differentially delivered from the two loudspeakers for a duration of 83 ms (35° from center). Because the loudspeakers were on the sides of the screen, and thus not exactly at the same position as the Gabor patches, cues were delivered in stereo from both speakers with different amplitudes in each channel so that sounds appeared to be coming exactly from the targets’ possible locations. Before the experiment was carried out, the audiovisual latency was measured using an oscilloscope. The thus measured 43 ms audio-visual offset, with no visually identifiable jitter, was taken into account in the stimulation program.

Participants were told that a vertical Gabor patch could appear in one of two placeholders, and they were requested to press a button as fast as possible when they detected its appearance in either of the placeholders. Participants were warned that in 1/3 of trials, no visual stimulus would appear on screen, and that auditory stimuli were non-predictive of target location. Auditory cues were absent in 1/5 of all trials, balanced across conditions. Fixation lasted for 500 ms before each trial. The trial started with the appearance of a circle around the fixation dot. Then a jittered amount of time would elapse (1–1.5 s) before target appearance. On 2/3^rd^ of the trials a Gabor patch was presented, half of them in the left and the remaining half in the right placeholder. This was followed by another jittered delay (1–1.5 s), then a response window of 1.5 s, during which the experiment waited for the participant to respond. If the subject pressed the response button at any time during the trial, their response would be recorded, and the experiment would move on to the following trial. The sound cues could be presented during either of the jittered delays, at various SOAs from the target: −600 and −150 in the pre-cueing conditions, +150 and +450 in retro-cueing conditions. All trial types were randomly intermixed within each experimental block.

Participants were asked to maintain fixation throughout each trial, and fixation was monitored online using the EyeTribe tracking device: if fixation was broken by more than 3° during the course of a trial, any response would be discarded and the trial be queued to be played again at the end of the block.

The experiment lasted for about 2 h and was divided into three parts. The first part was a practice block with feedback after each trial, which amounted to 1 block of 64 trials. The second part was a psychophysical staircase to find the appropriate level of contrast for each participant to reach ~70% accuracy overall. This staircase was performed separately for both visual hemi-fields after a pilot experiment showing significant differences in performance depending on which side the target appeared (1.1% ± 0.3 for right-sided targets, 0.9 ± 0.3 for left-sided targets at the beginning of experiment). The staircase block was conducted using the Psychtoolbox-3 Quest procedure^49^ on 3 blocks of 40 trials without feedback or auditory stimuli, using default parameters. This staircase was followed by 20 experimental blocks of 60 trials, for a total of 1,200 trials. These were evenly split among 5 experimental conditions: −600 ms, −150 ms, +150 ms, +450 ms and no-cue, each containing 240 trials. As this is a go-no-go task, 1/3 of trials in each category was “target-absent”. In each experimental condition, this led to 160 “target-present” trials, and 80 “target-absent” trials. However, when a cue and/or target is absent (i.e. less than two stimuli are presented), the notions of SOA and congruency are inapplicable because they refer to the relationship between two events. This eventually leads to there being 160 trials per SOA (further split between congruent and incongruent trials), plus a pooled number of 80 × 4 = 320 target-absent but cue-present trials, as well as 80 target-absent and cue-absent trials, for a total of 400 target-absent trials which belong to no SOA or congruency category.

Even though the staircase procedure was calibrated for a 70% performance at the beginning of the experiment, participants were told to aim for 75% performance, and we tried maintaining this performance level throughout by adjusting the Gabor patch contrast between blocks. This discrepancy was due to the fact that the staircase procedure was run without any auditory cues, and participants’ overall performance usually increased by about 5 points between staircase and test session. During the course of the experiment, the target contrast was readjusted by 0.1% contrast (applied on both left and right targets) when global performance on the preceding block was either too high (more than 80% hits) or too low (less than 70% hits).

### Analysis

#### Trial exclusion criteria

Trials were automatically discarded when the timings weren’t respected during the experiments (hardware failure). We also discarded all trials where the response time was under 150 ms after target onset on the assumption that these were artefactual: the minimum amount of time to produce a voluntary response is generally considered to be higher than this cutoff ^50^. This amounted to a loss of less than 3 ± 2% of trials on average. There was no upper RT boundary for trial exclusion because large RTs can drastically change the interpretation of data^51,52^. In particular, although distributions of RT data are positively skewed (see more details below), there is often significant information contained in the tail, and it is thus generally recommended not to truncate the tail of the distribution in order to make it resemble a normal distribution^52^.

#### Task accuracy and signal detection theory

Task accuracy was evaluated using signal detection theory^53^. We computed d’ (*Z*(hit rate) − *Z*(false alarm rate), the measure of unbiased sensitivity to a stimulus’ presence in all experimental conditions. We then performed a classical two-way repeated measures ANOVA on the obtained values, with Greenhouse-Geisser correction when required. Post-hoc pairwise comparisons were Bonferroni-corrected 10,000 bootstrapped confidence intervals of the mean difference between tested conditions.

#### Response time data and IES analysis

The analysis of response time data requires some amount of care: because RT values are always positive, they have a lower boundary, but no upper boundary. As such, RT distributions typically have a rightward skew, where the mean, mode and median differ from each other. As such, classical analyses are ill-fitted to analyze RT data due to the skewed nature of RT distributions^42,54,55^. Several alternative methods can be used to analyze distributions with a rightward skew. Some use appropriate parent distributions such as the shifted Wald^43^, gamma^50,51^, log-normal^56^, or ex-gaussian^57^ distributions, but the choice of model is rather arbitrary and the results should be interpreted with care^42,58^. We applied a generalized linear mixed-effects (GLMM) model^59^ to fit the Hit reaction time data and the inverse efficiency score (IES)^40,41^ as suggested in^42^. For IES analysis, each RT was divided by the average accuracy of the condition and participant it belonged to. The computations were performed using the lme4 library^59–61^ running in open-source software R^62^. The random effects of all models were not maximum^63^, as this led to convergence issues when running the procedure. We thus restricted them to a random effect of participant number on the intercept for all analyses. To define statistical significance of fixed effects, we used a backward stepwise simplification approach which helps find a model with only statistically significant fixed effects: we started with a full model including all factors and meaningful interactions (SOA, congruency and their interaction). At each simplification step we then calculated all models that differed from the current one by dropping a single term among fixed effects while maintaining the same random effects and compared these reduced models to the original one with a likelihood-ratio test^64^. We then excluded the terms that could be dropped without a significant increase in unexplained deviance as indicated by the likelihood-ratio tests and fitted a new model without these terms, thus obtaining the initial model for the next step. We repeated the procedure until no more terms could be dropped without a significant worsening of the model fit (as indexed by a significant increase of the residual deviance; the likelihood ratio tests of all terms were significant). The statistical significance of the terms in the final model was reported via a similar procedure for each term: a full final model with the effect in question was tested against a reduced model without the effect in question; we reported the p-value of the likelihood ratio test of this comparison^65^. Pairwise comparisons on GLMM models were performed when the fitted model included an interaction term, using the *multcomp* package in R^66^ and the appropriate vignette as a reference^67^. We performed two-tailed Tukey’s all-pair HSD multiple comparisons for planned contrasts (here, the effect of congruency at different SOAs) with a Bonferroni-Holm correction for multiple comparisons. Unplanned contrasts (the effect of SOA when compared to the no-cue condition) were assessed using 10,000 bootstrapped confidence intervals of the mean difference between tested conditions. These analyses were followed by a correlation analysis between effect sizes at the SOAs where a significant effect of congruency was present for the IES. The differences in IES between congruent and incongruent conditions were tested using Spearman’s correlation coefficient between relevant conditions.

#### Response time data modeling

The use of a GLMM with a 2-parameter inverse-Gaussian parent distribution is more appropriate for statistical testing than a classical 2-way ANOVA. However, the resulting estimated parameters are difficult to interpret in terms of cognitive processing. More complex analysis methods use models thought to describe the underlying process leading to the production of the timed response, by modeling signal accumulation. Among the most well-known is the Drift Diffusion Model first proposed by Ratcliff *et al*.^68,69^, which assumes that evidence accumulates towards a response following a random walk with a drift until the amount of evidence accumulated crosses a decision boundary, at which point the perceptual decision is taken and the corresponding response is prepared and executed. It is widely used to model the process underlying 2 alternative force-choice tasks with speeded responses, where the drift rate can be either positive (towards response A) or negative (towards response B). Here, we use a variation of this framework^43^ that has 3 parameters of interest (Fig. 2a): information accumulates at a rate γ, which depends on the quality of information accumulated, until it reaches the decision boundary α which depends on cognitive control, and the overall time taken to produce a response includes an additional non-decision time θ, which corresponds to all the time processes unrelated to decision-making take.

For response time data analysis, only Hits (correct response in a target-present trial) were analyzed. False alarm response times were not analyzed. We then modeled response time distributions using a shifted-Wald (or shifted Inverse-Gaussian, with 3 parameters) distribution and a maximum-likelihood estimation procedure from Anders *et al*.^43^. Drift-diffusion models are generally used to describe 2-AFC tasks, where there exists two competing response options. In our case however, the task is a go/no-go task, where participants opt-out of responding when they feel they did not see a target. In this case, the Wiener process, which is designed to model 2-AFC tasks, is inappropriate for modeling. Several of its parameters are poorly or non-applicable, in particular the estimation of how much bias each participant has towards one possible answer, which cannot be estimated in our case: when participants choose to opt-out, there is no response time data to be modeled. A good substitute for go/no-go tasks is the shifted-Wald (SW) distribution, which has already been used to describe go/no-go drift-diffusion processes in several studies^43,70,71^. The shifted-Wald distribution, also called the shifted inverse-Gaussian is of the form:

$$SW(RT|\gamma ,\alpha ,\theta )=\frac{\alpha }{\sqrt{2\pi {(RT-\theta )}^{3}}}\ast \exp \{-\frac{{[\alpha -\gamma (RT-\theta )]}^{2}}{2\,(RT-\theta )}\}$$

Anders *et al*.^43^ propose a Maximum Likelihood Estimation (MLE) fitting method fully described in their publication^43^. The basic rationale behind it is to minimize the difference between the observed RT data quantiles and the model-predicted quantiles using a single parameter β. From this β, all three parameters mentioned above can be directly calculated by closed-form maximum-likelihood estimators^72^ and used to compute the model-predicted quantiles for comparison and minimization in regards to the observed quantiles. We used their methods and adapted their analysis script to fit our needs.

In the analysis pipeline used, we fit a separate SW distribution to each experimental condition for each participant, and considered the global RT distribution to be a mixture of these. We only modeled response time distributions for experimental conditions where we observed an effect of the SOA or congruence, which were the short pre- and post-cued SOAs (−150 ms and +150 ms). With 20 participants, this led us to fit 2 (SOAs) × 2 (congruence) × 20 (participants) individual SW distributions, leading to as many estimations for each separate parameter. 2 × 2 ANOVAs and Bonferroni-corrected 10,000 bootstrapped confidence intervals of the mean difference were then conducted for significance testing.

## Supplementary information

Supplementary Information

## Data Availability

Data and scripts for modeling in R are available at https://github.com/DaphneRR/Retro-Pink.
