# Supplementary material for: Retrospective auditory cues can improve detection of near-threshold visual targets
Source: Sci Rep. 2019 Dec 12;9:18966. doi: 10.1038/s41598-019-55261-0 (PMC6908653; doi:10.1038/s41598-019-55261-0)
Supplement: Supplementary file 1 — Supplementary Information [file 41598_2019_55261_MOESM1_ESM.docx]

Retrospective auditory cues can improve detection of near-threshold visual targets

Supplementary Information

Daphné Rimsky-Robert^1^, Viola Störmer^2^, Jérôme Sackur^3^ and Claire Sergent^1^

^1^ Integrative Neuroscience and Cognition Center - UMR 8002 CNRS/Université Paris Descartes

^2^ Department of Psychology & Neuroscience Graduate Program, University of California, San Diego

^3^ LSCP, École des Hautes Études en Sciences Sociales


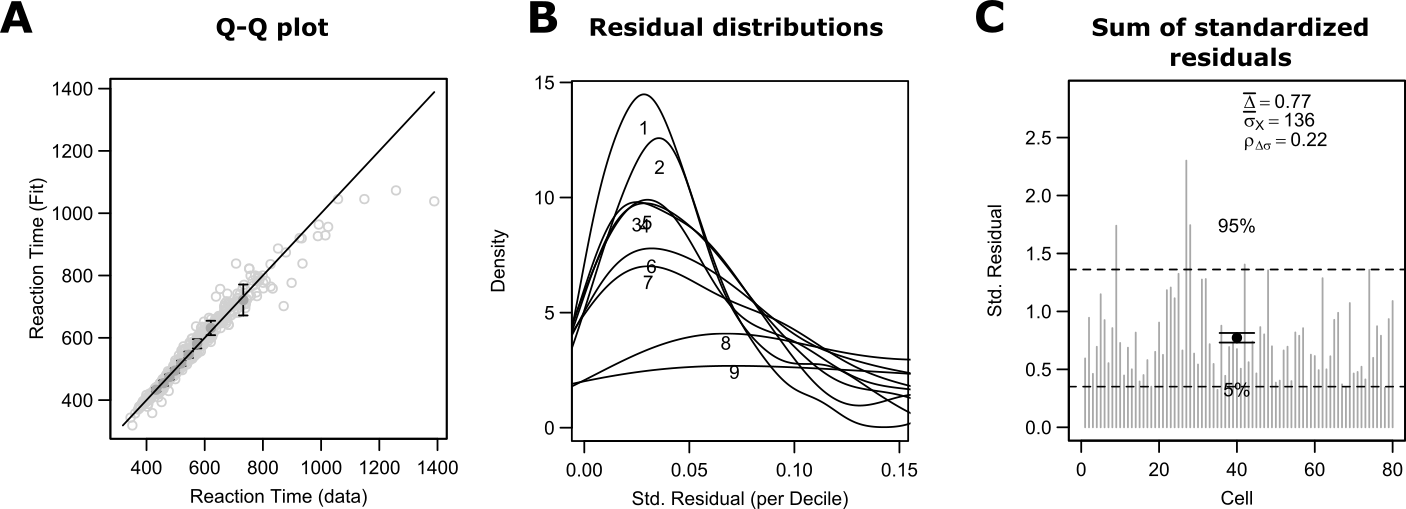


**Supplementary Figure 1: Model diagnostics**. The fitted RTs are plotted against real data. If the model fits the data appropriately, there should be no systematic deviation of these values from the diagonal. Here, there is no evidence of such deviation, but the plot indicates that some large RT values are outlying (**A**). Residual distributions are split by decile. These should be ordered, with a gradual increase in variance. Here, the distributions are ordered, with a larger variance than expected, which may be indicate of outliers in the last deciles (**B**). The sum of standardized residuals is expressed per “experimental cell”. The shifted-Wald distribution was fitted for each condition per participant, each of these is an “experimental cell”. These should have the lowest mean $\bar{\Delta}$and correlation to their standard deviation $\rho_{\Delta\sigma}$possible. Here, this assessment is fair, but not ideal (**C**). Overall, these diagnostics indicate the model acceptably fit the data.
